# Every dog has its day: a new journal for canine genetics and epidemiology

**DOI:** 10.1186/2052-6687-1-1

**Published:** 2014-04-16

**Authors:** William ER Ollier, Lorna J Kennedy

**Affiliations:** Centre for Integrated Genomic Medical Research, University of Manchester, Manchester, M13 9PT UK

The launch of a new journal dedicated to canine genetics and epidemiology is both timely, and needed, to support research communities within veterinary medicine, wildlife conservation, evolutionary biology and canine comparative health genetics. In this context, we are delighted to announce the launch of *Canine Genetics and Epidemiology*, an open access journal published by BioMed Central with the support and backing of the UK Kennel Club.

The last decade has witnessed an unprecedented increase in genetic analysis of canine species especially the domestic dog. This has largely been driven through major advances in molecular biology, high throughput DNA sequencing and genotyping, comparative genomics, statistical methodology and the development of high powered computers. Sequencing of the dog genome has been a critical factor for driving this progress. The importance of the dog, both to science and veterinary medicine, is underscored by the fact that its genome was one of the first mammalian species to be sequenced after man. Having established the dog genome sequence, we are now defining the level of genetic variability both within and between breeds, and investigating and refining the evolutionary relationships between canine species. Such knowledge is now having an impact on research, translating into clinical benefit through the introduction of new screening initiatives for disease related gene variants and also informing breeding programmes. Eventually genetic knowledge will help tailor and optimise therapeutic dosage and indicate which drugs should be used or avoided.

These advances are now leading to major discoveries, which need to be published as quickly as possible, and communicated to the widest possible audience. It can be difficult to publish canine genetic and epidemiological studies in specialist (mainly human) journals, as they are likely to be assessed by referees who are more experienced with human rather than canine research. Some aspects of study design for canine genetic analyses differ significantly from the way human population studies are currently investigated. This is because human populations tend to be mostly unrelated, in contrast to the dog, where within breeds, dogs are often closely related. Human disease association studies can require sample sizes of many thousands of cases and controls to generate the statistical power needed to detect genetic risk factors; in canine studies it has been shown that sample sizes of a hundred or fewer may suffice. Furthermore, statistical analyses used for human studies may be inappropriate for use in canine studies of inbred populations. Existing journals are often too general to assess canine genetic and epidemiology papers appropriately and may not have sufficient specialist referees since each journal receives a limited number of canine papers. Currently, it is rare to have rapid publication of canine genetics research.

Epidemiological studies exploring canine disease risk are now expanding rapidly, arising from analyses of data from second opinion referral centres and pet insurance companies. More robust approaches, such as the use of longitudinal cohort studies, are also being introduced. Reporting both canine genetic and epidemiology research reports in a single journal is appropriate, as both genetic and environmental factors contribute and interact to trigger disease processes. It is important that both genes and environment should not be studied in isolation. Through publishing both canine genetic and epidemiological research in a single journal, we hope to bring research communities closer.

Domestication of the dog from the wolf some 10–15,000 years ago, is a recent event in terms of evolutionary time scale. Sequencing genomes for different canine species is underway and is revealing their evolutionary relationships. This will lead to important insights into canine physiology, health and disease, and help facilitate conservation and wildlife management policies.

Comparative genetic approaches using studies in one species to help identify disease related genes in another species are well established. Comparative maps of the genomes of a wide range of species exist, and it is possible to cross reference the chromosomal position of a disease causing gene found in one species to its location in another species. The term “animal model” is often used to describe animal based pathology which represents that seen in human diseases; many of these models are experimentally induced rather than occurring naturally. Furthermore, many experimentally induced animal disease models represent only partial aspects of their counterpart human condition. The standard model species are usually mice or rats. However, the dog genome is more similar to that of the human than rodents. It is now recognised that domestic dogs spontaneously develop many clinical conditions that are also seen in humans, and these may have similar if not identical underlying pathological processes. Examples include cancers, autoimmune diseases, allergies, eye diseases, musculoskeletal and neurological conditions. Veterinarians involved in research benefit from specialist training to clinically describe their canine patients, unlike research scientists working with rodent animal disease models. The research potential that the dog offers for comparative studies has started to bring dog owners and breed clubs together with veterinary and human clinical scientists to develop collaborations which benefits both human and canine medicine.

The existence of a wide range of dog breeds where some are particularly predisposed to certain diseases represents great potential for conducting comparative studies. In many ways they are similar to isolated inbred human populations, such as the Amish, which have been critical for identifying disease causing mutations. Most domestic dog breeds are of recent origin having been developed over the last 300 years. Selective breeding of dogs with particular physical or behavioural attributes, have been fixed by restricting breeding to within defined pedigrees. Across all dog breeds there is now a wide range of gene mutations encoding the extremes of many physical and behavioural characteristics; e.g. the size variation between Great Dane and Chihuahua. One consequence of breed formation has been that undesirable and/or unrecognised disease causing genes have been inadvertently included along with desirable attributes.

The potential of the domestic dog for comparative disease studies formed the basis for a four year EU Frame Work 7 research award called LUPA. This brought together research geneticists, epidemiologists and veterinarians across Europe with the sole mission identifying the genetics of canine diseases that also represent important human conditions. A wide range of diseases were included.

Two aspects are seen to be paramount for the scope and focus of this new Journal. Firstly, that the Journal should be open access, so it can disseminate research and knowledge to a wide, international readership. Secondly that it should completely embrace “public engagement with science”. There are many dog owners and breeders around the world committed to understanding and improving the health and welfare of domestic dogs. Further potential readers include those who are committed to wildlife and conservation biology. This journal will provide an opportunity to bring scientists, veterinarians, dog owners and an interested general public closer together.

Every article published will have a lay summary and we will occasionally include lay interpretations of complex scientific terminology.

